# Supplementary material for: Barriers and facilitators to prudent antibiotic prescribing for acute respiratory tract infections: A qualitative study with general practitioners in Malta
Source: PLoS One. 2021 Feb 11;16(2):e0246782. doi: 10.1371/journal.pone.0246782 (PMC7877739; doi:10.1371/journal.pone.0246782)
Supplement: S1 File — (DOCX) [file pone.0246782.s001.docx]

**Supplementary file 1**

**Interview guide**

1. May you briefly tell me something about your work as a general practitioner?
2. How do you look upon the role of antibiotics in medicine?
3. How do you look upon antibiotic use and resistance in Malta (from a local and national level)?
   1. What factors do you think are contributing to this resistance in Malta?
4. What do you understand by the term ‘unnecessary antibiotic prescribing’?
5. Have you heard about ‘delayed antibiotic prescribing’?
   1. What do you understand by ‘delayed antibiotic prescribing’?
   2. What are your views on ‘delayed antibiotic prescribing’?
6. What are your general thoughts on the antibiotic prescribing practices of other Maltese GPs?
7. What are your views on the ‘drug rep culture’ in Malta?

*So now I would like you to reflect upon the last time you prescribed antibiotics and another time when you did not prescribe antibiotics for an acute respiratory tract infection. I would like you to respond to some related questions, keeping those scenarios in mind.*

1. What are your views on antibiotic prescribing for acute respiratory tract infections?
   1. Please describe your experience with antibiotic prescribing for acute respiratory tract infections.
   2. Please give me some examples of specific situations in which you prescribed or did not prescribe antibiotics for acute respiratory tract infections.
   3. How do you select which antibiotic to prescribe?
2. What kind of information do you provide your patients who present with an acute respiratory tract infection?
   1. How do you look upon your role as an educator?
3. Which factors do you think influence the way you prescribe antibiotics?
4. How do you feel your patients impact your antibiotic prescribing?
5. How do you feel the pharmaceutical industry/medical reps impact your antibiotic prescribing?
6. What are your thoughts on near-patient testing to aid in identifying infections?
   1. Do you make use of near-patient tests yourself?
7. What do you do if someone in your own family/a close relative has an acute respiratory tract infection?
8. What do you think are your specific needs in terms of antibiotic prescribing for acute respiratory tract infections?
9. Is there anything else that you would like to add?

***Thank you for your time and valuable insight into this topic!***
