# Supplementary material for: Barriers and facilitators to prudent antibiotic prescribing for acute respiratory tract infections: A qualitative study with general practitioners in Malta
Source: PLoS One. 2021 Feb 11;16(2):e0246782. doi: 10.1371/journal.pone.0246782 (PMC7877739; doi:10.1371/journal.pone.0246782)
Supplement: Attachment — Submitted filename: Review PLOS one 2020.docx [file pone.0246782.s002.docx]

Review PLOS one General practitioners’ understanding of antibiotic use and resistance, and perceived barriers and facilitators to prudent antibiotic prescribing: a qualitative study

Understanding antibiotic prescribing and resistance remains a very important topic to be explored and tailored interventions to the needs of the context are essential. Therefore this interesting article can contribute to the already existing literature but needs a little bit more work.

Abstract

Methods

Line 28: ‘quota sample’, what does this mean? How does this differ from purposeful sampling and how does this then link to concept of data saturation?

Same comment to line 118, what kind of sampling was used ? do you mean with quota sampling some form of purposeful sampling?

The interview data is already rather old as the interviews were done in 2014. It does not mean the data is not valuable anymore but it would be good to put it in context of that time in qualitative research especially in the discussion section and limitation.

Line 149: had the interviewer some experience in qualitative research? Has she been trained in interviewing?

Line 164 : what is the background of AN (as this might have an impact on the data analysis so it is good to know as a reader). Have both persons analysed all transcripts or only a few?

Results but linked with methods

There is an elaborate description of the many subthemes and then at the end there is a small description of the socio ecological model, I think in my personal opinion, as I was reading through the results I was already thinking that applying that model would have been useful on the data set. You do that at the end but maybe it would make the result section better if you organize it in that way from the beginning and explain this also in the method section, it will make the piece a lot stronger and easier to read.

Discussion

In my view, the description of the results again could be shorted a bit or maybe interpreted a little bit more as it is now a bit repetitive of the results section. Maybe it could be useful to start from the socio ecological model and link to the most important point that will need to be addressed in interventions. Also highlight what is new in your research compared to other research or what is more specific for Malta (as this is the reason why this research has been set up) than what you can find in the literature.

Methodological considerations

Starting from Line 908

“In qualitative research it is customary to use small, non-probability samples. We used quota

909 sampling, a strategy that considers sizes and proportions of subsamples, in order to obtain

910 subgroups that reflect corresponding proportions in the population103 and therefore attempt to

911 gain a more representative picture”

I would not start with defending the ‘small sample’ in qualitative research this is normal and we shouldn’t defend it but it is important to be reflective about your sample, who have you sampled, who haven’t you got sampled, what might you have missed,… to ensure quality of your data. Could you be a bit more reflective of your sample size, would there be potential participants that you might have missed? Data saturation is also linked to who you have selected for interviewing but also the interview questioning (the more structured the quicker you will get ‘saturation’ for example) but it is also linked to the experiences of the interviewer, how skilled are they? So it would be good to be a bit more reflective about that as well (and to mention also these skills in the method section) as these are (and some more) are essential criteria for saying we have data sufficiency.

I am not sure the aim in qualitative research is to get a representative picture for the whole population, but it is rather the aim is to find information rich cases to the phenomenon under investigation and personally I am more in favour of using the term ‘transferability’ to argue for the potential of wider relevance of the results which can be the result of the composition of the sample (i.e. diverse sample), rather than the sample size.
