# Supplementary material for: Barriers and facilitators to prudent antibiotic prescribing for acute respiratory tract infections: A qualitative study with general practitioners in Malta
Source: PLoS One. 2021 Feb 11;16(2):e0246782. doi: 10.1371/journal.pone.0246782 (PMC7877739; doi:10.1371/journal.pone.0246782)
Supplement: Attachment — Submitted filename: Response to reviewers_2021.01.16.docx [file pone.0246782.s004.docx]

| **Department of Global Public Health**  Erika A. Saliba-Gustafsson, PhD  Karolinska Institutet  Tomtebodavägen 18A,  171 77 Stockholm  Sweden | PLOS Carlyle House  Carlyle Road Cambridge, CB4 3DN United Kingdom |
| --- | --- |

**RE: Revision of manuscript entitled: “Barriers and facilitators to prudent antibiotic prescribing for acute respiratory tract infections: a qualitative study with general practitioners in Malta” [PONE-D-20-11324R1]**

Dear Dr Himmel,

Thank you for giving us the opportunity to revise our manuscript and for considering it publication in PLOS ONE. Thank you also to the you and the reviewer for taking the time to thoroughly critique our manuscript. Enclosed kindly find our responses to all comments received together with an updated version of our manuscript (both marked and unmarked). We made several new revisions to the manuscript, including restructuring the discussion and reducing the word count further.

We look forward to hearing back from you at your earliest convenience.

Kind regards,

**Erika A. Saliba-Gustafsson,** **PhD**

**Academic Editor Comments:**

1. I would recommend moving the last sentence of the Findings section in the Abstract (“Findings were collated … prescribing.”) to the end of the Methods section. And you should present 2 or 3 more results in the Findings section of the Abstract. I think you have room enough there and it makes the Abstract more interesting and this may stimulate readers to read the whole paper.

RESPONSE: We agree that it would be better to move the last sentence of our findings to the methods and have now done so. Thank you for pointing that out to us! We also presented additional findings in the abstract as recommended.

2. You talk about a “formative” study at the beginning of the Methods. I’m afraid this is an unusual description of your study type and/or irritating for readers since they typically know this expression form educational research (summative – formative assessment). Is “qualitative” not sufficient – what do you think?

RESPONSE: Thank you for pointing this out to us. To avoid any misunderstanding (and to further reduce the word count) we have now removed “formative” from the beginning of the Methods sections.

3. A better heading of Category III may be: “Why GPs use or do not use diagnostic tests”. I think it’s not so much “attitudes” that matter but structure (e. g., access to facilities, lack of resources and so on). Of course, your decision!

RESPONSE: Thank you for your comment. We have considered your recommendation and chose to revise the category to, “GPs’ views on diagnostic testing and its availability in Malta” (lines 252-253). We changed attitudes to views since the findings reflect GPs’ views of the situation in Malta, which is of course very much impacted by their lack of access to diagnostic testing facilities in this setting.

4. Perhaps “GPs’ experience in patient education” is a better heading for Category V (instead of “role”).

RESPONSE: Category V has now been rephrased to read “GPs’ experiences with patient education” (line 316).

5. Following Reviewer 1, my major concern refers to the structure of your Discussion section. Your socioecological model is really interesting but I was disappointed that you did not use this innovative approach to structure your discussion. Instead, you discuss several of your results, sometimes with a subheading, sometimes without (e.g., when discussing the role of pharmacists). For the reader, this selection of issues and the structure of how you present them must appear arbitrary. I strongly recommend to try the following: Take the subheadings of your socioecological model as subheadings of your Discussion and discuss (as short as possible!) how the interviews with the GPs inform this model or to put it the other way how the barriers and facilitators to prudent prescribing cannot be understood if we only look at GPs and their education/knowledge. The model helps us to consider the many other factors that influence antibiotic prescribing. I know that the first three areas or levels of the model are far better represented in your interview material than “community” and “public policy”, but, for example, guidelines and insurance do play a role in “public policy” for GPs, as you found out. So, please try to structure the discussion this way and get back to me if it is impossible.

RESPONSE: Thank you for your comment. We have taken your advice into consideration and restructured the discussion to highlight key issues whilst better reflecting the socioecological model. Since organisational-level and public policy-level factors have a great impact on diagnostic uncertainty, which is discussed in the paper, we chose to combine the two, splitting that sub-section into various headings relevant to those levels of the socioecological model.

6. If it is possible (and I do hope so!) you should report one important “limitation” of your study or study design. Even if the model helps to better understand antibiotic prescribing, your study only captures the model as seen/perceived by the GPs. Further research should examine the 5 areas of the model not alone from the GP perspective but as ‘realities’ of their own. Questions, for example, could be: Did information campaigns take place and did they change antibiotic prescriptions and so on. I think your paper could show how the future of research in drug prescribing could be and what elements are needed. Take the chance!

RESPONSE: Thank you for pointing this out. We have changed our methodological considerations to focus on strengths and limitations of the study, to include both limitations related to only including one perspective (the GPs’) but also to address reviewer #1’s comment (that the data dates to 2014). It is worth noting however, that little has changed since 2014 in this context, so our findings are still relevant.

**Reviewers' Comments:**

**Reviewer #1:**

Dear authors, well done on addressing comments adequately apart from comment 5 about the data been collected in 2014, so still valuable but it might already be a bit dated, so you need to address this in the strength and limitation section.

The discussion still needs a bit of work I think, as you have used the socioecological model, I would expect to find some findings and implications related to those different levels (intrapersonal, interpersonal, organisation and community and policy level) word count is still a lot, I personally find an article of more than 5000 words too lengthy and people might not read it. It is worth trying to get the essence said in under 5000 words.

RESPONSE: Thank you for your comments. We have done our utmost to address these concerns. Please see our responses below.

- We have included a strengths and limitations section that raises the issue of our data being collected in 2014.
- Discussion has been re-structured to reflect the socioecological model better.
- We noted your concern regarding the word count and reduced it further.
